# Diaphragmatic Rapid Shallow Breathing Index Versus Diaphragmatic Thickness Fraction for Predicting Weaning Success in Mechanically Ventilated Patients

**DOI:** 10.7759/cureus.116038

**Published:** 2026-09-10

**Authors:** Ritu Baloda, Monica Chhikara, Sahil Shiva, Sudha Puhal, Anju Rani, Neena Bhatti

**Affiliations:** 1 Anaesthesiology, Pandit Bhagwat Dayal Sharma University of Health Sciences, Rohtak, IND; 2 Anaesthesiology, Pandit Bhagwat Dayal Sharma Post Graduate Institute of Medical Sciences, Rohtak, IND; 3 Pharmacology and Therapeutics, Christian Medical College and Hospital, Ludhiana, IND

**Keywords:** critical care, diaphragmatic rapid shallow breathing index, diaphragmatic thickness fraction, diaphragmatic ultrasonography, mechanical ventilation, ventilator weaning

## Abstract

Introduction

Timely liberation from mechanical ventilation is a central goal of critical care. This study aimed to compare two point-of-care ultrasound indices, the diaphragmatic rapid shallow breathing index (D-RSBI) and the diaphragmatic thickness fraction (DTF), in predicting successful weaning among mechanically ventilated adults.

Methods

In this prospective, single-center diagnostic-accuracy study, 44 adults who were mechanically ventilated for more than 48 hours but fewer than seven days and who met the readiness criteria for a spontaneous breathing trial (SBT) underwent diaphragm and lung ultrasound before the trial. Weaning outcomes were categorized using the Weaning according to a New Definition (WIND) framework; successful weaning (simple or difficult categories, n = 36) was the reference standard, whereas prolonged weaning (n = 8) was considered the adverse outcome. Diagnostic performance was assessed at operating thresholds and by receiver operating characteristic (ROC) analysis, and the two indices were compared using the DeLong test (SPSS Statistics v23 (IBM Corp., Armonk, NY); MedCalc (Ostend, Belgium)).

Results

At a threshold of 1.2 breaths/min/mm, the D-RSBI showed strong diagnostic performance for successful weaning, with a sensitivity of 94.44% and specificity of 100% (area under the curve (AUC): 0.99, 95% confidence interval (CI): 0.97-1.00). The DTF discriminated poorly (sensitivity: 61.11%, specificity: 62.50%; AUC: 0.65, 95% CI: 0.42-0.88). The D-RSBI AUC was significantly higher than that of the DTF (DeLong p = 0.004). Notably, all eight patients with prolonged/failed weaning were also the eight most severely ill patients according to the Acute Physiology and Chronic Health Evaluation II score (APACHE II score, 13-21 vs. ≤ 10 in every patient who succeeded), and the direction of association for both indices was opposite to that reported in previous literature.

Conclusions

In this cohort, a D-RSBI ≥ 1.2 was strongly associated with successful weaning and outperformed the DTF. However, weaning outcome was completely determined by illness severity in this cohort, and the direction of the associations for both indices was inverted relative to prior studies. Therefore, these findings must be interpreted with caution and require validation in larger, multicenter cohorts.

## Introduction

Weaning from mechanical ventilation is a pivotal step in the care of patients with acute respiratory failure of respiratory, neurological, or postoperative origin. Delaying separation exposes patients to complications such as ventilator-induced lung injury and ventilator-associated pneumonia, whereas premature separation may precipitate aspiration, impaired airway protection, or recurrent respiratory failure [1]. Identifying the ideal time to wean remains difficult, and several bedside indices have been proposed to support this decision [2]. Point-of-care ultrasonography (POCUS) now allows clinicians to evaluate a range of dynamic and static markers of respiratory performance, including the diaphragmatic rapid shallow breathing index (D-RSBI), the diaphragmatic thickness fraction (DTF), diaphragmatic excursion (DE), and the lung ultrasound score (LUS) [2,3].

The diaphragm is the principal muscle of spontaneous respiration, and prolonged mechanical ventilation can rapidly impair its structure and function [4,5]. The Weaning according to a New Definition (WIND) framework categorizes weaning outcomes based on the number and timing of separation attempts: no weaning (no attempt), Group 1 (success at the first attempt), Group 2 (success within seven days of the first attempt), and Group 3 (success only after more than seven days following the first attempt or failure to wean) [1]. The primary objective of this study was to evaluate the association of D-RSBI) and DTF with weaning outcomes and compare their diagnostic performance in predicting successful weaning. The secondary objective was to assess the association between LUS and weaning outcomes.

## Materials and methods

Study design, setting, and participants

This prospective, single-center diagnostic-accuracy study was conducted in the ICU of a tertiary care teaching hospital. The study period extended from August 2024 to December 2024. The protocol was approved by the institutional Biomedical Research Ethics Committee (Pt. B. D. Sharma PGIMS/UHS, Rohtak; approval no. BREC/23/TH Anaesthesia/30; committee registration no. EC/NEW/INST/2022/HR/0189; dated October 17, 2023); the approval letter was provided as supporting documentation. We obtained written informed consent from every participant or their legally authorized representative. We prospectively registered the study with the Clinical Trials Registry of India (CTRI) under registration number CTRI/2024/08/072124.

We enrolled 44 adults (aged > 18 years) who had been ventilated for more than 48 hours but fewer than seven days and met standard criteria for a spontaneous breathing trial (SBT), including stable oxygenation and hemodynamics, minimal sedation, an effective cough, absence of fever, and a hemoglobin concentration of at least 8 g/dL [1,6]. Patients were excluded if they had received a neuromuscular blocking agent within the preceding 48 hours, were pregnant, or had a thoracostomy, neuromuscular disorder, cervical spinal cord injury, or an inadequate acoustic window. The sample size was estimated based on a single diagnostic-sensitivity parameter using the following formula:

$\displaystyle{\displaylines{n = Z2&times;Sn​&times;(1&minus;Sn​)​/d2&times;P}}$

Where Z = 1.96, expected sensitivity (Sn) = 0.784, prevalence (p) = 0.37, and absolute precision (d) ≈ 0.20, resulting in a calculated sample size of 44 patients [7].

Outcome definitions and reference standard

SBT success was defined as completion of the 30-minute trial without predefined signs of intolerance. The SBT was conducted for 30 minutes using pressure support (PS) of 8 cmH₂O, positive end-expiratory pressure (PEEP) of 4 cmH₂O, and a fraction of inspired oxygen (FiO₂) of 40%. SBT intolerance was defined by the presence of any of the following during the trial: respiratory rate > 35 breaths/min for more than five minutes, oxygen saturation (SpO₂) < 90%, heart rate > 140 beats/min or a sustained change of > 20% from baseline, systolic blood pressure > 180 mmHg or < 90 mmHg, or clinical signs of severe distress, including agitation, diaphoresis, or new-onset accessory muscle use. Following a successful SBT, extubation was performed if the patient met standard airway-protection and neurological criteria, specifically a Glasgow Coma Scale (GCS) score > 13 or return to baseline neurological status, an adequate cough reflex, and manageable airway secretions requiring suctioning no more frequently than every two hours.

SBT failure was defined as the inability to complete the 30-minute trial because of predefined signs of intolerance. Extubation failure was defined as the need for reintubation following separation from mechanical ventilation. Prolonged weaning was defined according to WIND Group 3 as a weaning process not successfully terminated within seven days of the first separation attempt, encompassing recurrent SBT or extubation failures. Weaning outcome was categorized according to the WIND framework [1] as simple (separation at the first attempt), difficult (success after up to three SBTs or within seven days), or prolonged (WIND Group 3). For the diagnostic-accuracy analysis, successful weaning - the WIND simple and difficult categories combined (n = 36) - was designated the reference-positive outcome, whereas prolonged weaning (WIND Group 3; n = 8, including two deaths) was designated the reference-negative outcome. This split was confirmed against the patient-level data.

Ultrasound assessment

Diaphragm and lung ultrasonography were performed with an ESAOTE ultrasound system (Esaote S.p.A., Genoa, Italy). All ultrasound examinations were performed with the patient positioned semi-recumbent at an angle of 30°-45°. To account for breath-to-breath variability, all diaphragmatic excursion and thickness measurements were recorded over three to five consecutive spontaneous respiratory cycles, and the resulting values were averaged for the final statistical analysis. The DTF was measured with a high-frequency linear probe (6-13 MHz) in the mid-axillary line between the sixth and 10th intercostal spaces. End-inspiratory and end-expiratory diaphragmatic thicknesses were recorded in M-mode, and DTF was calculated as [8]:

$\displaystyle{\displaylines{DTF = ​Tinsp​&minus;Texp​​/ Texp}}$

Where T_insp_​ is end-inspiratory diaphragmatic thickness, and T_exp_​ is end-expiratory diaphragmatic thickness. DTF was expressed as a fractional value, with 0.4 corresponding to 40% thickening [8].

Diaphragmatic excursion was obtained with a low-frequency curvilinear probe (1-5 MHz) subcostally along the mid-clavicular line; an excursion below 1 cm was taken to suggest dysfunction [3].

The D-RSBI was calculated as:

$\displaystyle{\displaylines{D-RSBI = RR/DE​}}$ Where RR is respiratory rate in breaths/min, and DE is diaphragmatic excursion in mm. The resulting D-RSBI was expressed in breaths/min/mm [9].

The LUS was calculated by scoring 12 lung regions, with each region assigned a score from 0 to 3 according to the degree of aeration loss, resulting in a total score ranging from 0 to 36 [10,11]. The physician making the extubation decision was blinded to all ultrasound findings. All ultrasound examinations were performed by two trained operators with seven years of experience in critical-care ultrasonography using a standardized acquisition protocol. Each examination was performed independently by both operators, and the mean of their measurements was used for analysis. Because raw operator-level measurements were not retained for independent statistical comparison, an intraclass correlation coefficient (ICC) could not be calculated. A D-RSBI threshold of 1.2 breaths/min/mm and a DTF threshold of 0.4 were pre-specified in the study protocol and used for categorical analyses; receiver operating characteristic (ROC) analysis was additionally performed. Illness severity was assessed using the Acute Physiology and Chronic Health Evaluation II (APACHE II) score [12].

Statistical analysis

Data were analyzed using SPSS version 23 (IBM Corp., Armonk, NY) and MedCalc (Ostend, Belgium). Continuous variables are summarized as mean ± standard deviation (SD), whereas categorical variables are presented as frequencies and percentages. Categorical associations were tested using the chi-square test and, because several strata had expected counts below five, Fisher’s exact test, including the Fisher-Freeman-Halton test for larger tables. Sensitivity, specificity, predictive values, and accuracy were computed against the reference standard with 95% confidence intervals (CIs). Discrimination was assessed using the area under the ROC curve (AUC) with a 95% CI calculated by the DeLong method, and the D-RSBI and DTF AUCs were compared using the DeLong test for paired curves. Reporting was conducted according to the Standards for Reporting Diagnostic Accuracy Studies (STARD) 2015 guideline [13]. The study flow is shown in Figure 1.

**Figure 1:**
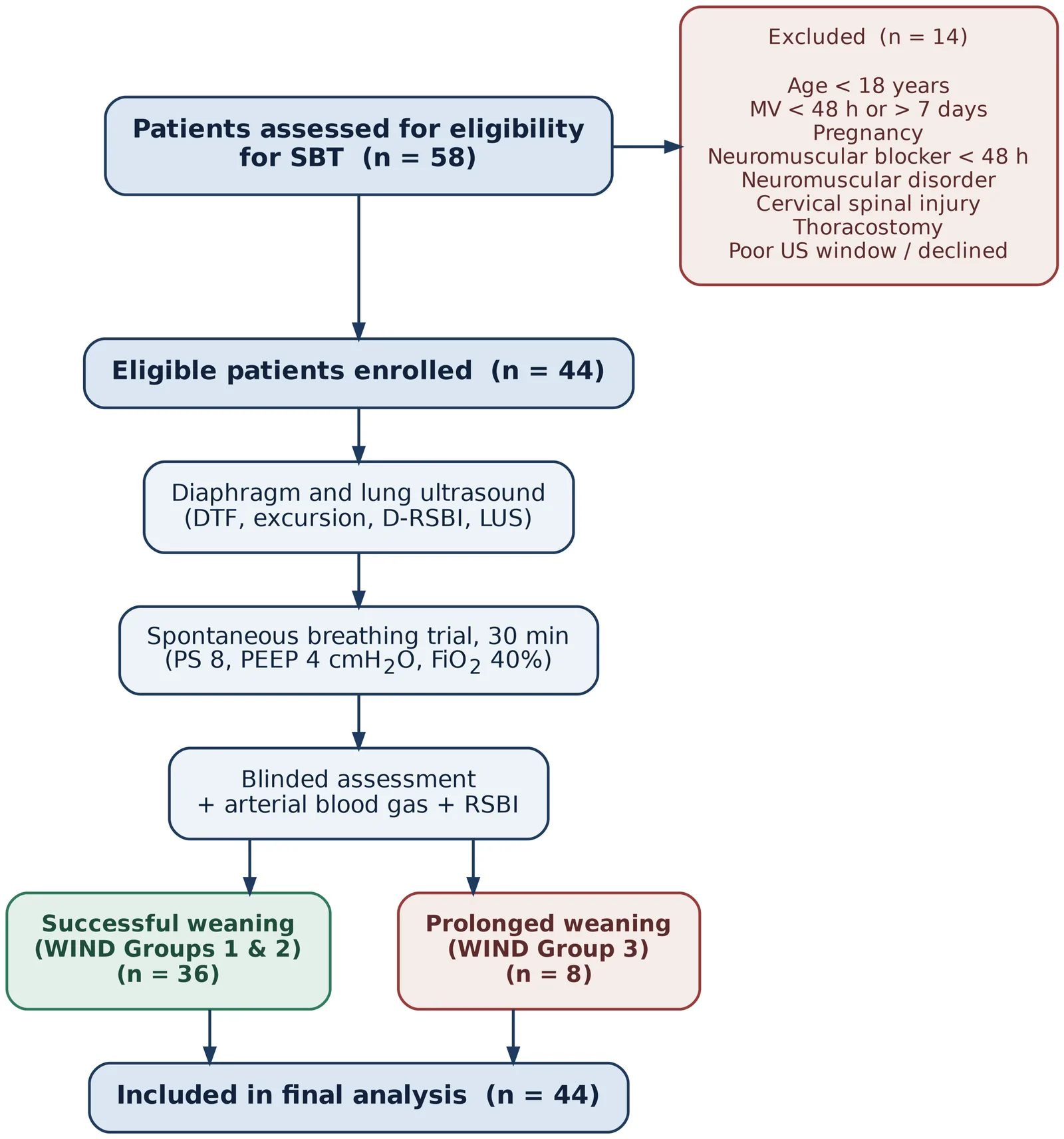
Study flow diagram Of 58 patients assessed, 14 were excluded, and 44 were enrolled and analyzed; 36 achieved successful weaning (WIND simple or difficult), and eight had prolonged weaning (WIND Group 3) D-RSBI: diaphragmatic rapid shallow breathing index; DTF: diaphragmatic thickness fraction; LUS: lung ultrasound score; MV: mechanical ventilation; PS: pressure support; PEEP: positive end-expiratory pressure; FiO₂: fraction of inspired oxygen; RSBI: rapid shallow breathing index; US: ultrasound; SBT: spontaneous breathing trial; WIND: Weaning according to a New Definition. Credit: Ritu Baloda

## Results

The cohort comprised 44 patients (mean age: 43.75 years; 40.91% aged ≤ 30 years; 54.55% women; Table 1). Respiratory distress was the most common indication for ventilation. The mean APACHE II score was 8.25 ± 4.74 (range: 4-21); 36 patients (81.8%) had scores ≤ 10, seven had scores of 11-20, and one had a score above 20 (Table 2). Weaning was classified as simple in 16 patients, difficult in 20, and prolonged in eight. Thus, successful weaning (simple + difficult) was achieved in 36 of 44 patients (81.8%).

**Table 1:** Baseline demographic characteristics of the study population (N = 44)

Characteristic	Frequency	Percentage
Age group (years)		
≤ 30	18	40.91%
31–50	10	22.73%
51–70	11	25.00%
> 70	5	11.36%
Sex		
Female	24	54.55%
Male	20	45.45%

**Table 2:** Distribution of the study population by APACHE II score (N = 44) APACHE II: Acute Physiology and Chronic Health Evaluation II; SD: standard deviation

APACHE II score	Frequency	Percentage
≤ 5	14	31.82%
6–10	22	50.00%
11–20	7	15.91%
> 20	1	2.27%
Mean ± SD: 8.25 ± 4.74; range: 4-21		

At a threshold of 1.2 breaths/min/mm, a D-RSBI value at or above 1.2 was present in 34 patients, all of whom weaned successfully, whereas eight of the 10 patients with values below 1.2 had prolonged weaning. This yielded a sensitivity of 94.44%, specificity of 100%, positive predictive value of 100%, negative predictive value of 80%, and accuracy of 95.45% (Tables 3, 4), and the association was significant with both chi-square and Fisher’s exact tests (χ² = 33.25, Fisher’s exact p < 0.001; Table 3). In the ROC analysis, the D-RSBI AUC was 0.99 (95% CI: 0.97-1.00).

**Table 3:** Distribution of D-RSBI, DTF, LUS, and APACHE II categories across weaning groups (N = 44) P-values were obtained using the Fisher-Freeman-Halton exact test (preferred because several strata had expected counts below five); chi-square (χ²) values are provided for reference D-RSBI: diaphragmatic rapid shallow breathing index; DTF: diaphragmatic thickness fraction; LUS: lung ultrasound score; APACHE II: Acute Physiology and Chronic Health Evaluation II

Parameter/category	Simple, n (%)	Difficult, n (%)	Prolonged, n (%)	χ²	Fisher's p
D-RSBI (breaths/min/mm)					
< 1.2 (n = 10)	1 (10.0)	1 (10.0)	8 (80.0)		
≥ 1.2 (n = 34)	15 (44.1)	19 (55.9)	0 (0.0)	33.25	< 0.001
DTF					
< 0.4 (n = 25)	11 (44.0)	11 (44.0)	3 (12.0)		
≥ 0.4 (n = 19)	5 (26.3)	9 (47.4)	5 (26.3)	2.17	0.377
LUS					
Abnormal (2, 3) (n = 5)	1 (20.0)	4 (80.0)	0 (0.0)		
Normal (0, 1) (n = 39)	15 (38.5)	16 (41.0)	8 (20.5)	2.92	0.251
APACHE II score					
≤ 10 (n = 36)	16 (44.4)	20 (55.6)	0 (0.0)		
11–20 (n = 7)	0 (0.0)	0 (0.0)	7 (100.0)	44	< 0.001
> 20 (n = 1)	0 (0.0)	0 (0.0)	1 (100.0)		

**Table 4:** Predictive validity of the D-RSBI (threshold 1.2) for successful weaning (N = 44) CI: confidence interval; ROC: receiver operating characteristic; D-RSBI: diaphragmatic rapid shallow breathing index

Parameter	Value	95% CI (lower)	95% CI (upper)
Sensitivity	94.44%	81.34%	99.32%
Specificity	100.00%	63.06%	100.00%
False-positive rate	0.00%	0.00%	36.94%
False-negative rate	5.56%	0.68%	18.66%
Positive predictive value	100.00%	89.72%	100.00%
Negative predictive value	80.00%	44.39%	97.48%
Diagnostic accuracy	95.45%	84.53%	99.44%
Area under the ROC curve	0.99	0.97	1

The DTF, dichotomized at 0.4, showed only moderate diagnostic performance (sensitivity: 61.11%, specificity: 62.50%, accuracy: 61.36%; Table 5), was not significantly associated with the outcome (Fisher’s exact p = 0.26; Table 3), and had an AUC of 0.65 (95% CI: 0.42-0.88). The D-RSBI AUC was significantly higher than the DTF AUC (DeLong test, p = 0.004; Figure 2). The 2 × 2 contingency tables underlying these estimates are given in Table 6.

**Table 5:** Predictive validity of the DTF (threshold 0.4) for successful weaning (N = 44) CI: confidence interval; ROC: receiver operating characteristic; DTF: diaphragmatic thickness fraction

Parameter	Value	95% CI (lower)	95% CI (upper)
Sensitivity	61.11%	43.46%	76.86%
Specificity	62.50%	24.49%	91.48%
False-positive rate	37.50%	8.52%	75.51%
False-negative rate	38.89%	23.14%	56.54%
Positive predictive value	88.00%	68.78%	97.45%
Negative predictive value	26.32%	9.15%	51.20%
Diagnostic accuracy	61.36%	45.50%	75.64%
Area under the ROC curve	0.65	0.42	0.88

**Table 6:** 2 × 2 cross-tabulation of each index against the reference standard (successful weaning), N = 44 Test-positive orientation reflects the direction observed in this dataset (D-RSBI ≥ 1.2 and DTF < 0.4 associated with successful weaning). This orientation is opposite to that reported in prior literature (see Discussion) D-RSBI: diaphragmatic rapid shallow breathing index; DTF: diaphragmatic thickness fraction

Index result	Successful weaning (n = 36)	Prolonged weaning (n = 8)	Total
D-RSBI (Fisher's exact p < 0.001)			
≥ 1.2 (test positive)	34	0	34
< 1.2 (test negative)	2	8	10
DTF (Fisher's exact p = 0.26)			
< 0.4 (test positive)	22	3	25
≥ 0.4 (test negative)	14	5	19

**Figure 2:**
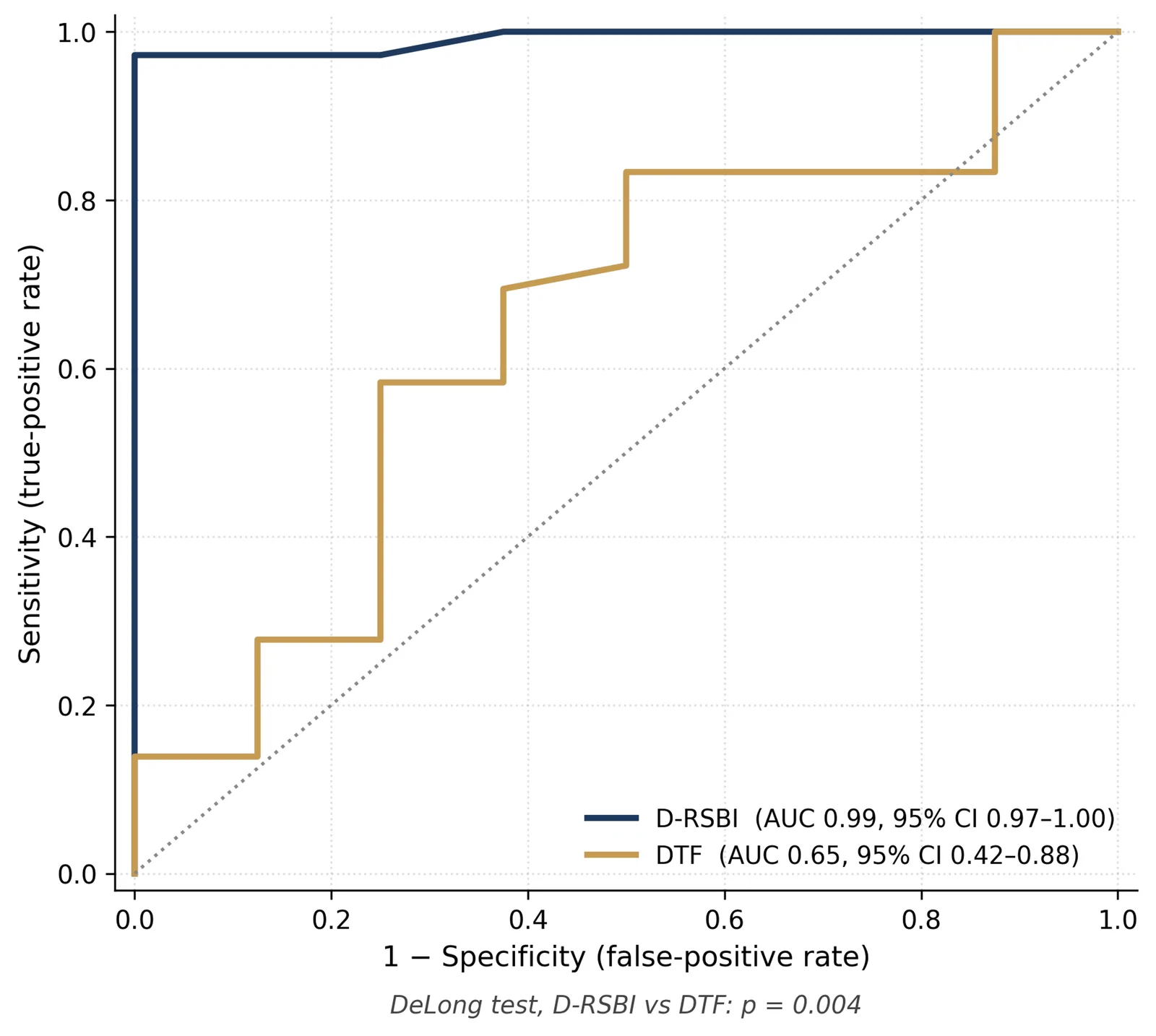
ROC curves of the D-RSBI and the DTF for predicting successful weaning The D-RSBI AUC (0.99) was significantly greater than that of the DTF (AUC 0.65) on the DeLong test (p = 0.004). Score orientation follows the direction observed in this dataset ROC: receiver operating characteristic; AUC: area under the curve; CI: confidence interval; D-RSBI: diaphragmatic rapid shallow breathing index; DTF: diaphragmatic thickness fraction

Lung aeration was not associated with weaning outcome: of the five patients with an abnormal LUS, one underwent simple and four underwent difficult weaning (with none experiencing prolonged weaning), whereas 79.49% (31/39) of those with a normal LUS achieved simple or difficult weaning (Fisher’s exact p = 0.25; Table 3). The conventional RSBI did not demonstrate significant predictive value.

Weaning outcome was closely linked to illness severity. All eight patients with prolonged weaning had an APACHE II score above 10 (range: 13-21), whereas every patient who weaned successfully had a score of 10 or below (mean APACHE II score of 17.5 in the prolonged group vs. 6.2 in the successful group; χ² = 44.0, Fisher’s exact p < 0.001). APACHE II score thus completely separated the two outcome groups.

## Discussion

In this single-center cohort, a D-RSBI at or above 1.2 breaths/min/mm was strongly associated with successful weaning (AUC: 0.99) and discriminated significantly better than the DTF (AUC: 0.65; DeLong p = 0.004). These diagnostic-performance estimates apply specifically to the present cohort and should not be interpreted as evidence of independent predictive value or clinical utility. Because APACHE II completely separated the successful and prolonged-weaning groups, the independent predictive contribution of D-RSBI beyond illness severity cannot be established from this dataset. Neither the DTF, the LUS, nor the conventional RSBI was significantly associated with outcome. Taken at face value, these results suggest that the D-RSBI has strong diagnostic performance for identifying successful weaning in this cohort [14]. Two features of the data, however, require the results to be interpreted with considerable caution.

First, weaning outcome in this sample was completely separated by illness severity as measured by APACHE II. Every one of the eight patients with prolonged/failed weaning had an APACHE II score above 10 (range: 13-21), and two of them died, whereas every patient who weaned successfully had a score of 10 or below. Because APACHE II scores completely separated the two outcome groups, the apparent predictive performance of the D-RSBI may be substantially confounded by illness severity in this cohort. The apparent performance of the D-RSBI cannot, in this dataset, be disentangled from the severity of illness.

Second, and importantly, the direction of association for both ultrasound indices was opposite to that reported in the literature. Established work indicates that a lower D-RSBI predicts successful weaning. For example, Mowafy and Abdelgalel identified a cut-off of D-RSBI < 1.6 breaths/min/mm at 30 minutes, with lower values indicating success [15], with concordant findings reported across other critically ill cohorts, including patients with acute exacerbations of chronic obstructive pulmonary disease [16-18]. Similarly, a higher DTF (better diaphragmatic thickening) predicts success [7,14]. In the present data, the opposite was observed: higher D-RSBI values (≥ 1.2) and lower DTF values (< 0.4) were associated with success.

Because the objective outcome coding is secure (prolonged weaning included two deaths), this inversion is unlikely to be an outcome-labeling error; it more plausibly reflects a systematic issue in the acquisition or derivation of the ultrasound indices (for example, the units or the ratio used to compute the D-RSBI, or the thickening calculation for the DTF) and/or the severity confounding described above. The D-RSBI and DTF derivations should be re-audited against the raw excursion and thickness measurements before these findings are considered for use in clinical practice.

A genuine advantage of diaphragm ultrasound is that it offers an immediate bedside appraisal of respiratory-muscle function without radiation and complements clinical judgment when readiness is uncertain [6,14]. The conventional RSBI and the LUS were not predictive here, consistent with the view that a static measure of aeration alone may not forecast extubation success in a relatively young ICU population [6,19]. Reported DTF thresholds also vary widely between studies (commonly 0.28-0.4), reflecting differences in case mix and technique [7,14].

This cohort was relatively young (mean age 43.75 years; 40.91% ≤ 30 years) with a low comorbidity burden and was predominantly female (54.55%), in contrast to the older, sicker, male-predominant populations of several previous series [7]. These characteristics, the modest single-center sample, the operator dependence of ultrasound, and, above all, the complete confounding by illness severity and the inverted direction of the indices are important limitations that constrain the conclusions that can be drawn.

Larger, multicenter studies enrolling more heterogeneous ICU populations, with prespecified thresholds, adjustment for illness severity (for example, multivariable models including APACHE II), and audited ultrasound-derivation protocols, are needed to establish whether the D-RSBI adds independent predictive value for weaning. Serial diaphragm ultrasound during the SBT and integrated models combining the D-RSBI, DTF, LUS, and conventional indices also warrant evaluation.

Strengths

The study used a prospective design with a standardized ultrasound protocol and evaluated both the D-RSBI and the DTF in the same patient cohort. Weaning outcomes were classified using the WIND framework, and diagnostic performance was assessed using sensitivity, specificity, predictive values, accuracy, and ROC analysis.

Limitations

This study was conducted at a single center with a small sample size, including only eight patients with prolonged weaning, which limits the precision and generalizability of the findings. The small number of prolonged-weaning events also leads to imprecise diagnostic-performance estimates. Importantly, APACHE II scores completely separated the successful and prolonged-weaning groups, representing a major limitation and making it difficult to determine the independent predictive value of the ultrasound indices beyond the effect of illness severity. Ultrasound measurements were obtained at a single time point, limiting the assessment of changes during the weaning process. Although two operators independently performed the ultrasound assessments, raw operator-level measurements were not retained for independent statistical comparison; therefore, an ICC could not be calculated, and the absence of a formal assessment of interobserver agreement remains a limitation. The findings, including the observed direction of association between D-RSBI and DTF, should therefore be interpreted cautiously, regarded as hypothesis-generating, and validated in larger, multicenter cohorts.

## Conclusions

In this cohort, D-RSBI demonstrated superior diagnostic performance compared with DTF for identifying successful weaning. However, the complete separation of outcomes according to APACHE II scores and the small number of prolonged-weaning events preclude the establishment of independent predictive value or clinical utility. Given the observed reversal in the direction of association compared with previous literature, these findings should be considered hypothesis-generating and warrant validation in larger, multicenter cohorts.

## Appendices

Appendix 1: case record form

**Table 7:** Patient information

Field	Entry
Name	
Age/Sex	
CR No.	
Case No.	
Diagnosis	
Indication for ventilation	
APACHE II score	
Mode of ventilation	
Tidal volume	
Respiratory rate (RR)	
Pressure support (PS)	
PEEP	
FiO₂	
Duration of ventilation	
Temperature	
Blood sugar	
Complete blood count	
GCS	
RASS score	
RSBI	
DTF	
D-RSBI	
LUS	
Weaning outcome	

**Table 8:** SBT assessment

Parameter	First SBT		Second SBT (if applicable)		Third SBT (if applicable)	
	Before	After 30 min	Before	After 30 min	Before	After 30 min
Heart rate						
Blood pressure						
Respiratory rate						
GCS						
ABG						
PaO₂/FiO₂						
EtCO₂						

**Table 9:** Additional SBT assessments

Parameter	Fourth SBT (if applicable)		Fifth SBT (if applicable)		Sixth SBT (if applicable)		Seventh SBT (if applicable)	
	Before	After 30 min	Before	After 30 min	Before	After 30 min	Before	After 30 min
Heart rate								
Blood pressure								
Respiratory rate								
GCS								
ABG								
PaO₂/FiO₂								
EtCO₂								

**Table 10:** Weaning and outcome details

Parameter	Details
Cause(s) of termination of SBT	
Number of separation attempts until successful SBT	
WIND criteria	
WIND Group	☐ Group 1 ☐ Group 2 ☐ Group 3
Date of extubation	____ / ____ / ______
Noninvasive ventilation (if any)	☐ No ☐ Yes: __________________
Date of reintubation (if any)	____ / ____ / ______
Number of ICU days	__________________
Date of discharge from ICU	____ / ____ / ______
Death/adverse outcome	☐ No ☐ Yes: __________________
